# A Multiscale Topographical Surface Analysis of High Entropy Alloys Coatings by Laser Melting

**DOI:** 10.3390/ma16020629

**Published:** 2023-01-09

**Authors:** Maxence Bigerelle, Anaïs Galliere, Yucelys Y. Santana, Hervé Morvan, Mirentxu Dubar, Jean-François Trelcat, Laurent Boilet, Emmanuel Paris

**Affiliations:** 1 Laboratoire d’Automatique, de Mécanique et d’Informatique Industrielles et Humaines—LAMIH, University Polytechnique Hauts-de-France, CNRS, UMR 8201, 59313 Valenciennes, France; 2 Belgian Ceramic Research Centre, 7000 Mons, Belgium; 3 Bruker Nano Surface Division, 7 rue de la croix Martre, 91120 Palaiseaux, France

**Keywords:** multiscale topographical surface analysis, roughness, focus variation, HEAs coatings

## Abstract

High Entropy Alloys (HEAs) coatings obtained by laser melting (LM) technique were studied through a multiscale topographical surface analysis using a focus variation microscope. The laser melting creates a multiscale topography from under-powder size (incomplete or complete powder melting) to upper-powder size (process conditions). The surface topography must be optimized because of the significant influence on friction and material transfer during sliding wear. The analyses were shown that different pre-melting zone interactions were present. Statistical analysis based on covariance analyses is allowed to highlight the different process melting scales. The best LM parameter values to minimize Surface Heterogeneity were laser power (Pw) of 55 W, laser exposition time (te) of 1750 µs, and distance between two pulses (dp) of 100 µm.

## 1. Introduction

Coatings are used for a long time to protect from wear, corrosion, and other environmental attacks. Some of them are toxic, like hard chromium, because to obtain this coating, the electrolyte baths commonly use hexavalent chromium, which is toxic to humans [1]. The High Entropy Alloys (HEAs) are being used to manufacture coatings. Yeh et al. [2] defined the HEAs as a mix of at least five elements in an equimolar ratio with an atomic concentration going from 5 to 35 % in 2004. Cantor et al., [3] developed multi-component alloys in 2004 by induction melting pure elements in equal atomic proportions, among which the most significant is a five-component Fe_20_Cr_20_Mn_20_Ni_20_Co_20_ alloy that forms a single FCC solid solution with dendritic solidification. Therefore, using HEAs like coatings on finished elements appears as an alternative for industrial applications.

Ye X. et al., [4] studied the Al_x_CoCrNiCuFe coating deposited by laser cladding in 2011. They obtained HEAs coating nanostructured with BCC and FCC crystal structure, finding the addition of aluminum elemental promoted the transition of FCC to BCC structure. Additionally, Ye Q. et al., [5] studied HEAs coating with a nominal composition of CrMnFeCoNi fabricated by laser surface alloying in 2017; they have found that this coating shows excellent properties with a combination of corrosion resistance, ductility, and strength. 

Several other studies on different coating compositions were published for HEAs coatings by laser cladding like AlCoCr_x_FeNi [6], Al_2_CrFeNiMo_x_ [7], CoCrBFeNiSi [8], AlCoCrFeNiTi_x_ [9], and FeNiCoAlCu [10]. The laser cladding technique obtains thick coatings of about 1–5 mm. In addition, it was reported in other techniques to obtain HEAs coatings such as mechanical alloying [11], plasma spraying [12], high-velocity oxy-fuel (HVOF) thermal spray [13], and Physical Vapour Deposition (PVD) [14], among others. 

The manufacture of HEAs bulk was possible through the selective laser melting (SLM) technique [15]. This technique is currently used for the powder bed fusion technique of metal additive manufacturing, which allows for obtaining functional components with high structural integrity at a low cost and is compatible with various materials [16]. This technique can be used for large surfaces and thicknesses. Thus, the coatings can be synthesized with laser melting from mono-elementary high-purity powder is cheaper, less energetic, and faster. However, there is a challenge to the surface: we want to control the melting quality.

It is widely known that the SLM produces surfaces with high roughness, and generally, the parts manufactured by this technique require a post-finishing process. The commonly reported roughness values (Ra) for components manufactured by SLM are mostly between 10 μm and 20 μm, depending on the material, the processing parameters of the SLM, and the orientation of the construction [17,18,19,20]. Tonelli et al. [17] evaluated the influence of laser energy on the surface roughness for manufacturing CoCr alloy by SLM in 2020. They found that low energy (50–100 W) applied to the powder bed produces surfaces with high roughness (max R_a_ = 13 µm), while at high laser energies (>150 W), the surfaces are smoother (R_a_ = 2.5–3 µm). Several authors [17,21,22] agree that when low laser energy is used, the flow of the melt is unstable, producing an incomplete wettability and dispersion of the melt during the process; this leads to a discontinuous morphology.

Furthermore, it was found that during this process, spatter ejection of the molten material from the pool could be produced, causing cavities and roughness. The ejection of splashes is because the laser plume tends to expel the molten material from the liquid pool. These expelled particles solidify before falling onto the bed of powder and react with the oxygen in the atmosphere, which would change its composition, for example, by forming oxides [17]. Therefore, when the deposition parameters are not correctly selected, the unmelted powders and large drops may be found that solidify quickly and are larger than the diameter of the laser spot. These large forming drops are extremely undesirable and are also known as the balling process [17,21,23,24,25].

Wang et al. [19] also studied a CoCr alloy in 2017 obtained by SLM, finding that the number of particles expelled rises as the laser energy increases. In addition, it has classified the splashes into three types of morphology (spherical splashing, coarse spherical splashing, and irregular splashing) according to their origin either from recoil pressure, Marangoni effect, and heat effect in the molten pool. They have also shown that the ejected particles have almost the same initial composition but higher O, Si, and C contents.

Another problem found in the SLM process is the denudation of the substrate, which is the depletion of the powders in the regions surrounding the laser track [17,19,25,26]. These authors agree that the above phenomena are strictly related to the process parameters. The stability of the melting pool is dominated by the balance between splash ejection due to the laser plume and Marangoni convection, which tends to propagate the molten material from the center of the melting pool to the outer regions.

Researchers have analyzed these alloys to obtain better mechanical properties [27]. Coatings are the link between a piece and an external environment. Thus, it is also part of the piece which submits more degradation: corrosion and wear. The surface topography of coatings affects the behavior of piece in-service conditions. Many peaks and valleys corresponding to high roughness will directly impact tribological properties like wear resistance. During the sliding wear of rough surfaces, the peaks will have a higher concentration of stress, producing breakage and generation of wear particles. Therefore, if there is a high roughness, the coatings will be worn quickly and heterogeneously. 

This work was used selective laser melting techniques to obtain HEAs coatings composed of FeCrAlMnMo. Laser melting surface treatment promotes a non-homogeneous surface area. Tribological properties are influenced by surface roughness. Therefore, it becomes essential to assess the topography of the surface and analyze a large part of the piece coated. At the same time and with a unique measurement, we want to observe the roughness peaks (µm order) and the general roughness of the piece (cm order). These requirements imply finding a new method with a considerable inspection space, an acceptable resolution, and fast analysis. 

We suggest characterizing melting quality by measuring and analyzing surface topography. However, the quantitative surface quality measurement requires a high amplitude measurement over a relatively wide spatial range of investigation. The Brüker society has innovated a new focus variation apparatus to solve this metrology demand. This bench can observe topography (with a lateral scale from 0.3 µm to 80 mm and a vertical scale from 0.01 µm to 300 µm) without filling in the blanks with outliers. It is a common problem for other optical measurement techniques with steep grades of roughness. The measure processing associated with multi-scale analysis highlights a topographical parameter. This parameter quantifies the melting quality for manufacturing HEAs coating quality, i.e., the part of the surface to be subjected to a total transformation. Therefore, with the focus variation methodology, we can create a multi-scale morphological indicator that quantifies the microtextural homogeneity of the HEAs coating manufacturing.

## 2. Experimental Process

### 2.1. Materials and Methods

**Figure 1:**
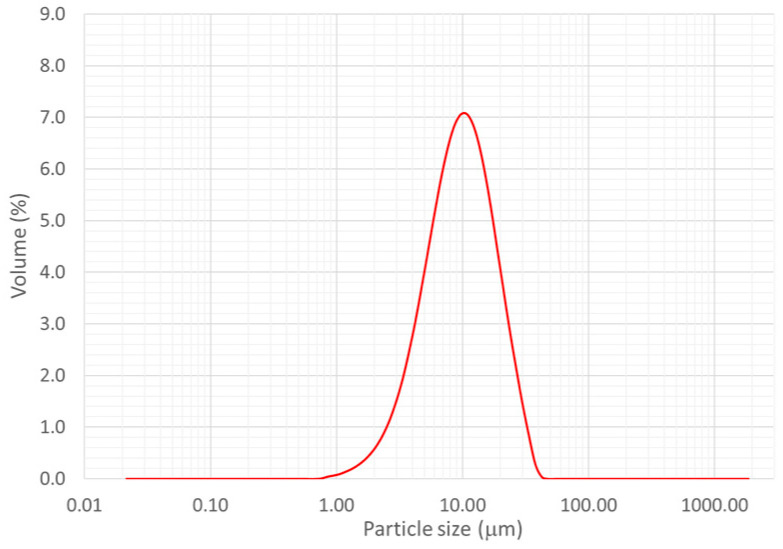
Diagram of the powder diameter repartition of the initial mix FeCrAlMnMo showing the grain size of the commercial mono-elementary powder after mixing.

The powders used to deposit the HEAs coatings were prepared with an ink of a mix of five mono-element industrial powders (>99% of purity) of Alpha Easar (Haverhill, MA, USA). The average granulometry of elements was for Fe: <10 µm, Al: 7–15 µm, Mn: <44 µm, Cr: <44 µm, and Mo: 3–7 µm. The curve in Figure 1 shows the grain size distribution of the mix realized from commercial mono-elementary powder, to confirm the grain size is more homogeneous after mixing.

The ink is 80% by mass of dry matter (i.e., 80% wt. powder/20 wt. water). The mixing of the metallic powders, water, and various organic materials (binder, dispersant, and antifoam) was conducted via a simple propeller agitator for 30 min. For the ink spray-coating, two layers are successively applied at a flow rate of 3 mL/min and with a constant gun speed of 100 mm/s.

**Figure 2:**
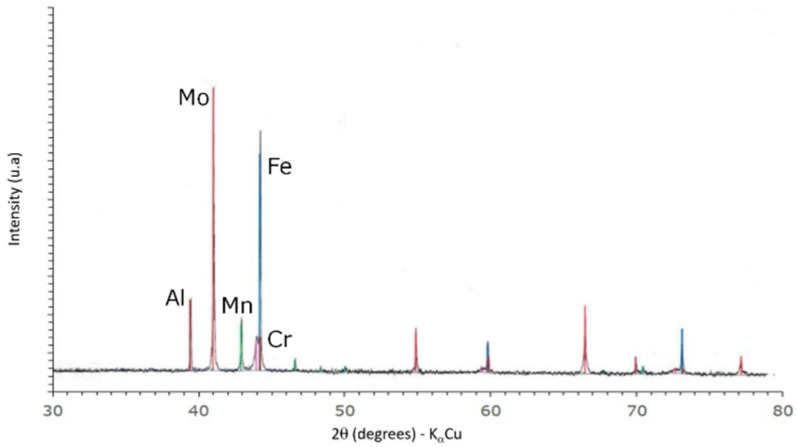
X-ray diffraction pattern of the initial powders FeCrAlMnMo after mixing, evidencing the presence of elemental components.

Figure 2 highlights that, before laser melting, any mono-element powder interacted with another. Five different peaks show corresponding metallic elements, which are like monophasic crystals.

The HEAs coatings were deposited on a steel substrate (S235JR) by a spray-coating technique after treatment with Laser Melting (LM). The ink is created by mixing powders (initial mixing: Fe_28_Cr_22_Al_20_Mn_19_Mo_11_) with aqueous and organic solutions. The ink is spread with a thickness of 100 µm, and the melting is performed with a SLM machine by two orthogonal passes of the laser. 

**Figure 3:**
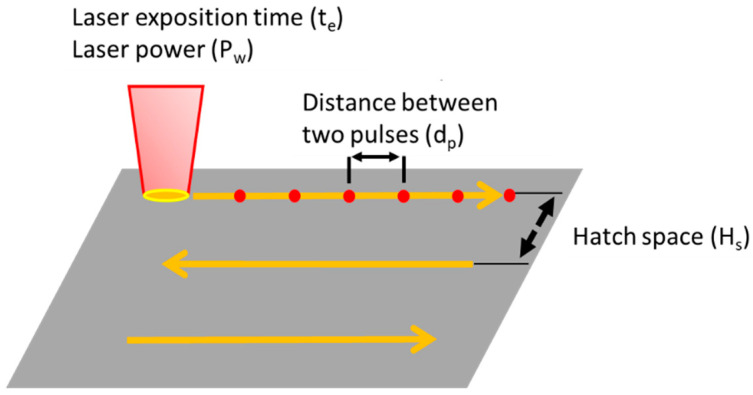
Schema of the different laser melting parameters selected to variate.

The SLM machine used in this study was a RenishawAM125 (Renishaw, Wooton-under-Edge, Gloucestershire, UK). It uses a high-power (Pmax = 200 W (cw)) fiber laser (λ = 1070 nm) and comprises a building chamber of 125 × 125 × 100 mm^3^ size, which is swept by argon to maintain an inert gas environment. The main parameters for fine-tuning processing are illustrated in Figure 3: Laser power (P_w_); Laser exposition time for each point (t_e_); (d) hatch space (HS), defined as the distance between two consecutive parallel laser tracks; and (e) distance between two pulses (P_d_), defined as the distance between two consecutive laser spot area irradiated. Table 1 summarizes the deposition conditions.

Morphological characterization and chemical composition were analyzed by scanning electron microscopy (SEM) and energy dispersive spectroscopy (EDS). The phase constituents of the coating were also analyzed by X-ray diffraction (XRD) with Cu target radiation (λ = 0.154060 nm). Hardness and reduced elastic modulus were determined by nanoindentation test with a diamond Berkovich indenter, using a Hysitron Triboindenter (TI980, Eden Prairie, MN, USA). The profile tests on the cross-section of the coating were performed in a mode of quantitative ultra-high-speed mechanical property mapping (XPM). This technology can perform six measurements/s. For each coating, three indentation arrays of 10 × 10 were carried out to a load of 2 mN. The spacing between indentations was 2 μm (i.e., 10–20 times the indentation depth) [28]. The hardness and elastic modulus were determined using the standard Oliver and Pharr method [29]. 

### 2.2. Topographic Analysis

**Figure 4:**
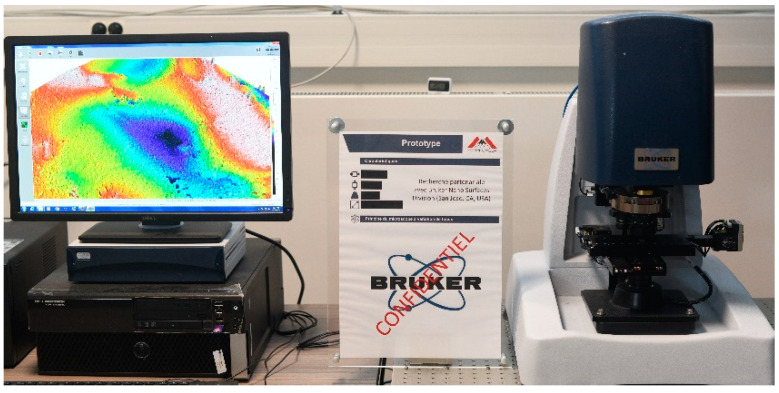
Image of focus variation microscopy (Contour LSK) developed.

The surface topographic study was realized using a focus variation microscopy of Bruker (Contour LSK, Bruker, Eden Prairie, MN, USA). Using a focus variation microscopy (Figure 4) allows acquiring very rough surfaces that other technologies (interferometric microscopy) do not permit obtaining [30].

**Figure 5:**
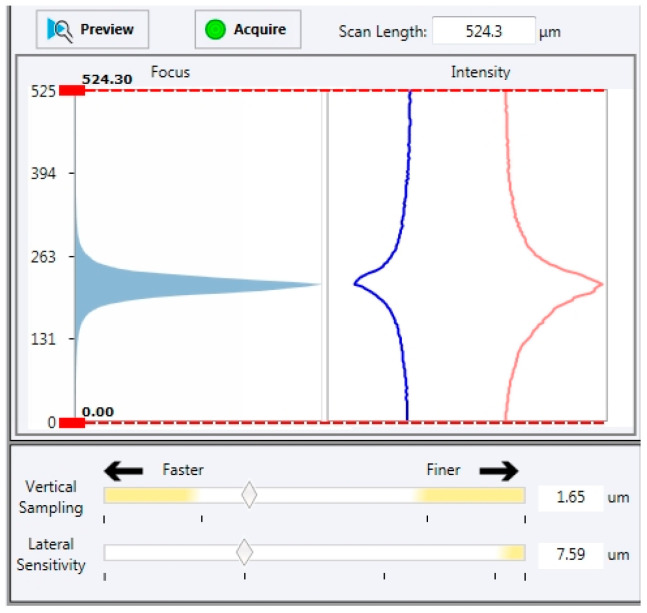
Contour LSK Bruker Interface. Histogram on the left represents the focus information curve. On the right, blue curve represents the lower intensity of pixels and the red one the higher intensity.

In most optical techniques, the lateral resolution of the lenses is a limiting factor in the measurement. It is especially true when measuring surfaces with steep slopes; there is a rapid loss of lateral resolution (sparrow criteria). The focus variation technique is different and uses both the lens properties (lateral resolution of the lenses and minimal depth of field) and the lateral sensitivity of the camera. The topography computation principle calculates the standard deviation of the grey levels of the image acquired in small local areas. Being based on image analysis, light management in the focus variation is strategic in acquiring topographic information. Therefore, Bruker developed tools that allow the user to know more or less the saturation quality of the CCD camera. This graphical interface allows the user to modulate the sample lighting by using the light ring for indirect illumination or the coaxial light for direct illumination (Figure 5).

The graphical interface gives access to relevant topographic information where other techniques fail due to a lack of information or are much slower. The use of nearby neighbors for height determination provides a more efficient resolution. This technique associates a map of standard deviations of grey levels between close neighbors (quality map), informing the user of the relevant topographic result obtained. The Bruker prototype gives a topographic map without bias to ensure topographical signal richness. Thus, it is possible to filter or modulate the quality map according to the scales we want to reveal for the study later. Then, the stitching method is used to widen the topographic field of view. This mathematical technique assembles the individual topography into a single global one. It creates a wide field of view while maintaining a high spatial resolution. A wide topographic field is obtained to analyze all spatial scales.

**Figure 6:**
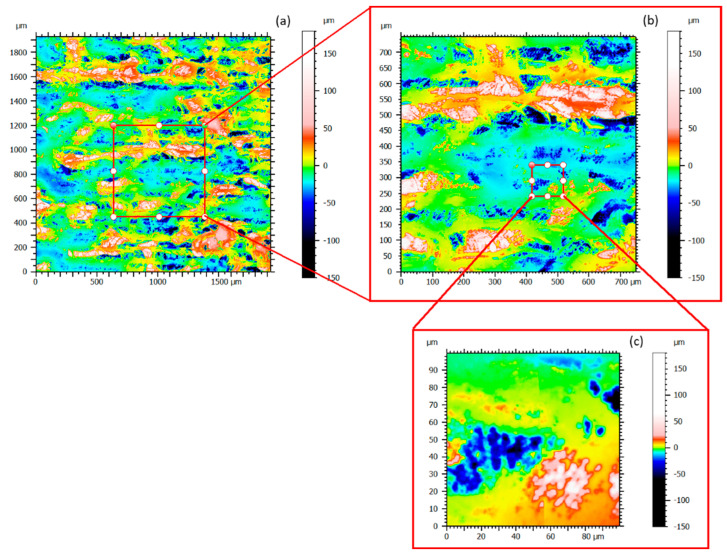
Stitching 2 mm × 2 mm in one topography measurement (**a**) Original topography Map; (**b**) X2 Zoom; (**c**) X20 Zoom.

Figure 6a shows a topography measurement by stitching 2 mm × 2 mm from the contour LSK Brüker. Such a critical measure gives a general state of the sample’s surface (ripples, large clusters, bumps, and shape, etc.). Although this surface measurement is enormous, some zones stand out like droplets around a hundred or a few micrometers in diameter. Zoom in a bump zone is performed and the extracted zone of 0.75 mm × 0.75 mm (Figure 6b) can still be studied thanks to the precision of the apparatus. A new topographical image is highlighted on this first zoom (Figure 6b). The big clusters that appear first as homogeneous are finally a bump with clusters on the surface: they are not smooth. Therefore, to try to understand what is shown exactly, another zoom is conducted and the extracted zone measured as 100 µm × 100 µm (Figure 6c). Instead of the enormous scale change, the topography obtained is still being studied. When the last extracted zone (Figure 6c) is compared to the initial topography without any zoom (Figure 6a), some clusters become visible, whereas they were almost invisible before extracting a smaller zone. The asperities were measured successfully, but for a more general observation (a few mm^2^), they were not observed because of the scale difference between the stitched surface and the smaller element.

## 3. Results and Discussion

### 3.1. Characterization of the Coatings

**Figure 7:**
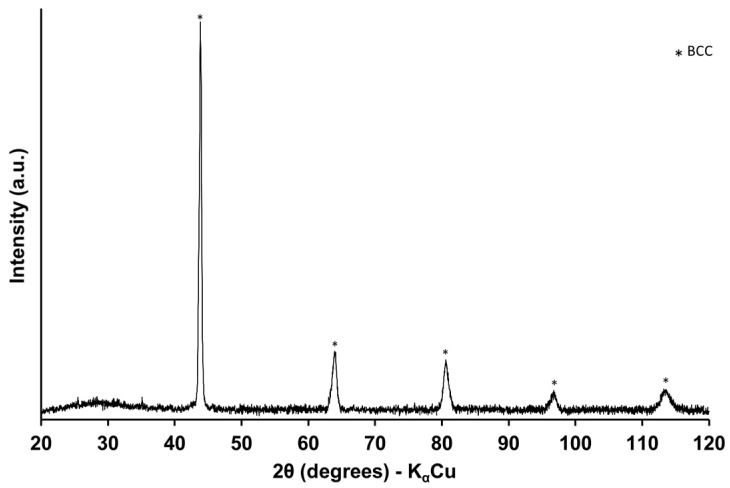
XRD of the selective laser melting HEAs coating.

An X-ray diffraction analysis was also carried out on the surface of the samples. Figure 7 presents a representative spectrum of the coatings; the structure consists of a single-phase BCC. 

**Figure 8:**
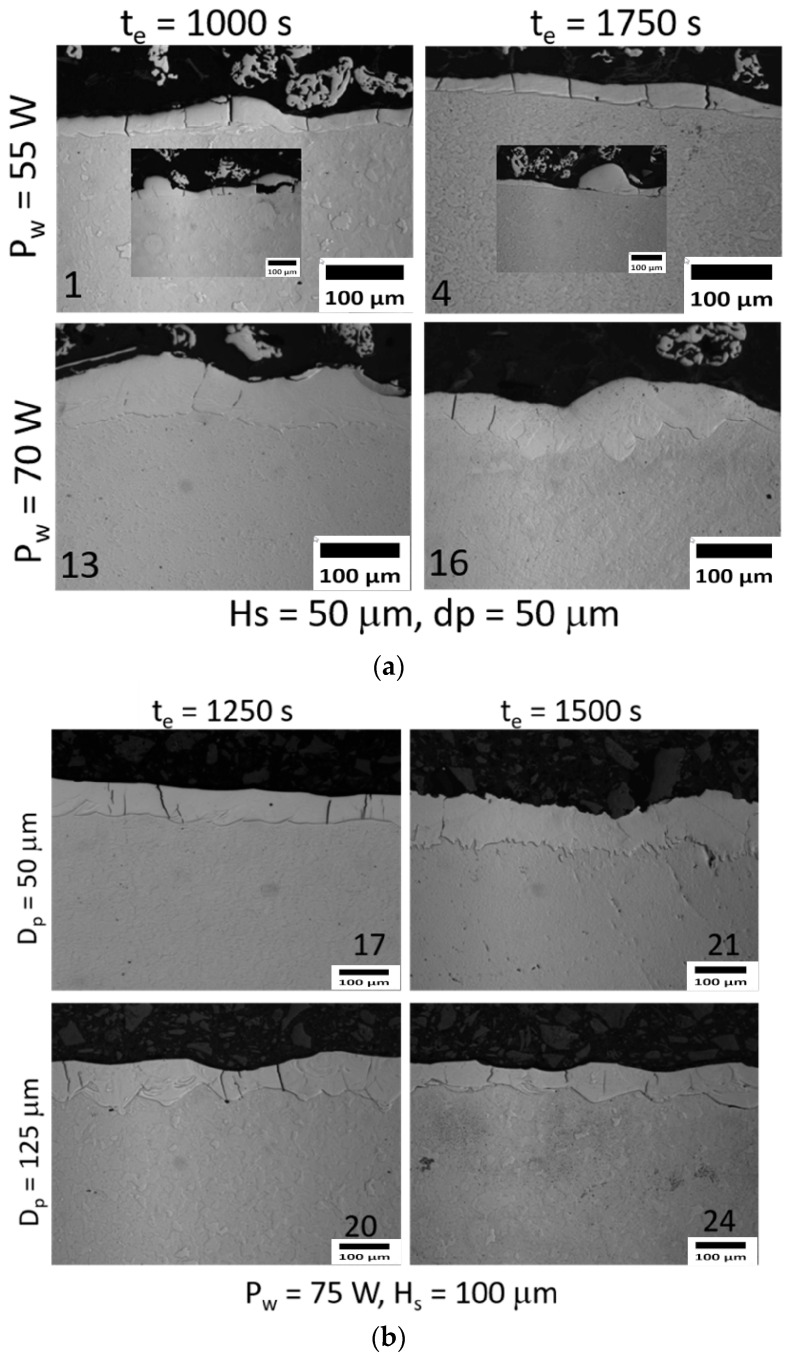
Images of the cross-section of coatings obtained by optical microscopy. The number (**a**) for H_s_ = 50 µm and d_p_ = 50 µm (**b**) P_w_ = 75 W and H_s_ = 100 µm.

**Figure 9:**
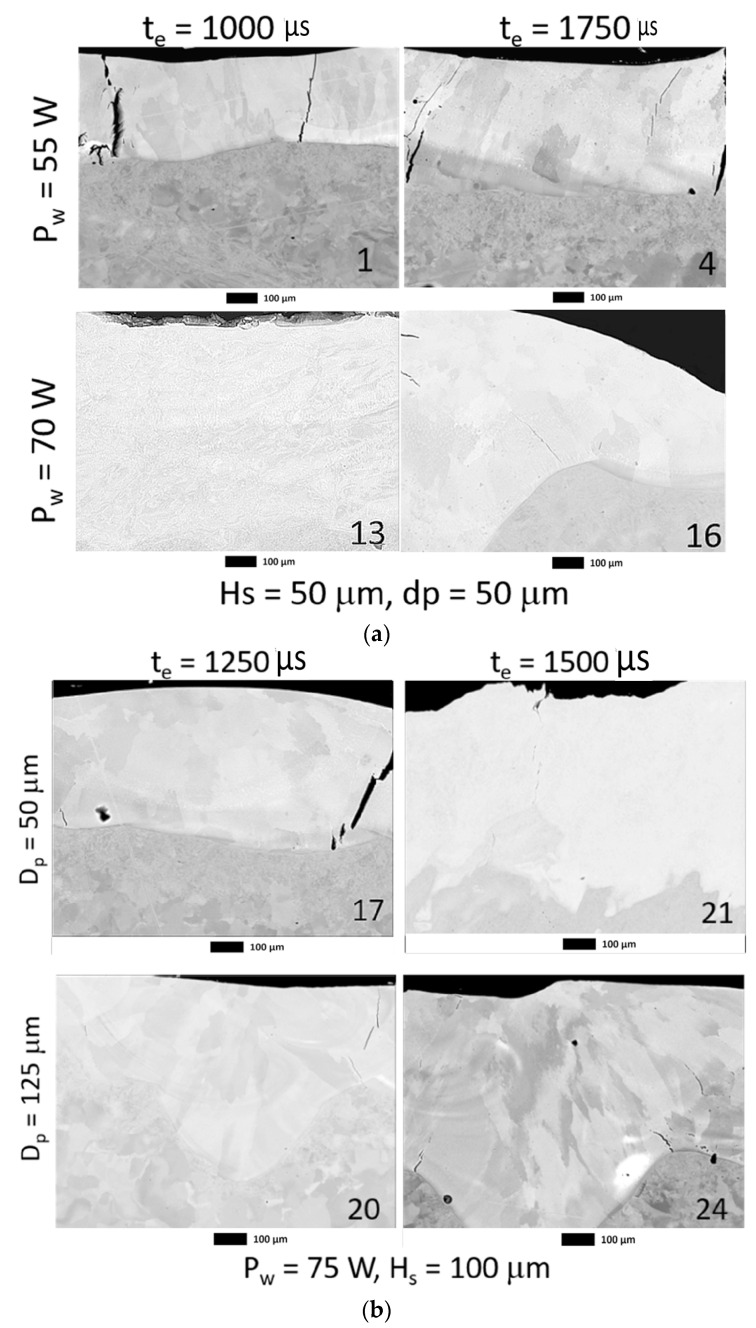
Images of the cross-section of coatings obtained by SEM. (**a**) for H_s_ = 50 µm and D_p_ = 50 µm (**b**) P_w_ = 75 W and H_s_ = 100 µm.

The coatings were characterized through the cross-section, and the extreme deposition conditions were selected. The chemical analyses by EDS are shown in Table 2 and Table 3 and the images obtained by optical microscopy (OM) and scanning electron microscopy (SEM) are shown in Figure 8 and Figure 9. For all the conditions observed in Figure 8, cracks were found perpendicular to the surface of the coating, these were in smaller quantities for the conditions of higher laser power (Hs and Dp of 50 μm). In addition, the coatings’ thickness, measured by OM image analysis at 100×, presented more influence on the laser power. Accordingly, a slight increase from 55 W to 65 W was observed with approximate values between 30 μm and 50 μm, and then at 70 W the thickness was more than double with high variation (80 μm and 90 μm). For the conditions deposited at 55 W denudation of the substrate and balling effect were observed, as can be shown in the micrographs of Figure 8. This phenomenon has been studied by different authors [17,21,23,24,25]. 

**Figure 10:**
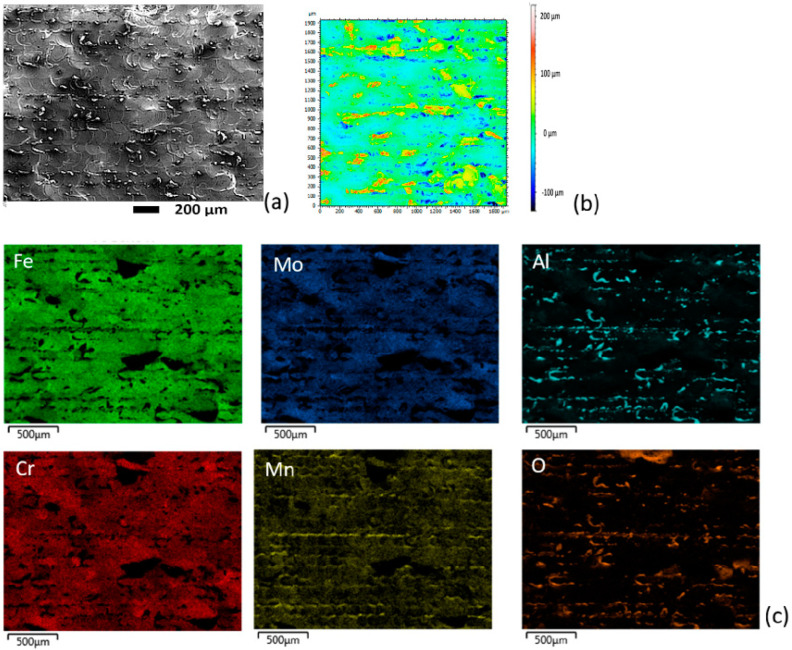
Surface analysis of the sample N° 24 for the laser melting HEAs coating. (**a**) SEM micrography, (**b**) topographical measure (**c**) EDS analysis.

Furthermore, the surface morphology of the coatings was observed by SEM and a representative micrograph of the coating for the sample N° 24 is presented in Figure 10a. It shows the presence of irregular splashing oriented in a direction that could correspond to the direction of the laser’s path, such as was found by Wang et al. in 2017 [19] and Tonelli et al. in 2020 [17]. It was widely studied that the laser melting technique produces highly rough surfaces due to the formation of drop spatter that then solidifies. The EDS mapping shows that these irregular droplets on the surface of the coatings contain aluminum and oxygen, which could correspond to aluminum oxide (Figure 10c). As explained by Wang et al. in 2017, the spatter powder can change its composition, for example, by reacting with oxygen. 

It was also evidenced in the EDS analysis (Table 2 and Table 3) that the chemical composition of the coatings changed respecting the initial powder mixing Fe_28_Cr_22_Al_20_Mn_19_Mo_11_. Therefore, the coatings were enriched in iron and depleted in the other elements as the laser power increased, possibly due to the dilution of the alloying elements in the substrate. 

**Figure 11:**
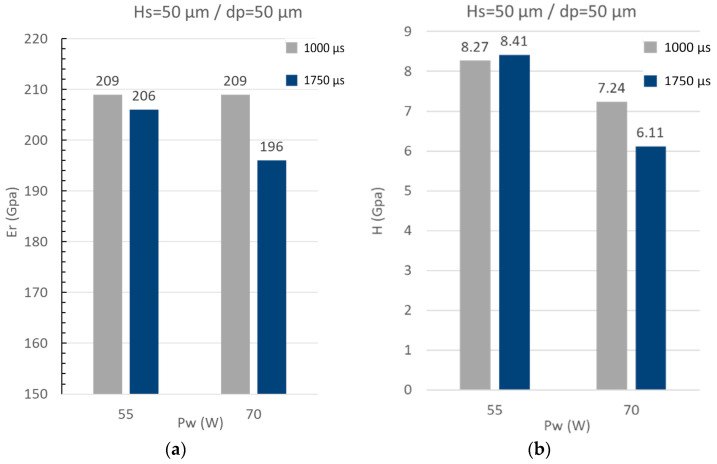

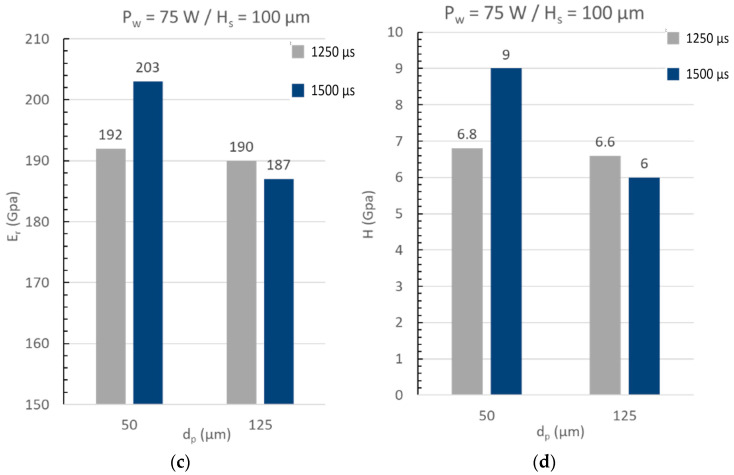
Graphs of hardness and reduced elastic modulus for different conditions of coatings. (**a**) Young modulus E_r_ for H_s_ = 100 µm and d_p_ = 50 µm with two laser exposition time of 1000 µs and 1750 µs; (**b**) Hardness H for H_s_ = 100 µm and d_p_ = 50 µm with two laser exposition time of 1000 µs and 1750 µs; (**c**) Young modulus E_r_ for P_w_ = 75 W and H_s_ = 100 µm with two laser exposition time of 1250 µs and 1500 µs; (**d**) Hardness H for P_w_ = 75 W and H_s_ = 100 µm with two laser exposition time of 1250 µs and 1500 µs.

The mechanical properties are determined for the extreme conditions (i.e., 55 W and 70 W) of laser power (Figure 11a,b) and the coatings obtained at 75 W for the distance between two pulses (d_p_) of 50 μm and 125 μm (Figure 11c,d). The results of hardness for the coatings obtain at 55 W of laser power were approximately 8 GPa and for 70 W varied between 6 and 7 GPa. The standard deviation for all measures was 0.5 GPa, therefore, it could be observed that the hardness decreases with the laser power possibly due to the diminution of elements such as Al, Mo, and Mn and an increase of Fe, such as have been referenced by other work [31]. The laser exposition time (te) has not shown significant variations in the hardness. Moreover, when the laser power was constant at 75 W, the hardness was approximately 6 ± 0.5 GPa for all conditions except for the coatings deposited with a d_p_ of 50 μm where the hardness was ~9 Gpa. It was supposed that the apparent grain size for this coating was smaller compared with the other conditions, as can be observed in Figure 9b for sample 21. The reduced elastic modulus determined for all conditions variated between 187 and 209 GPa with standard deviations between 5 and 10 GPa, therefore, it was not found that the variations of depositions parameters influence this property. 

The topographical image for these coatings (Figure 10b) shows the droplets, which generate a high surface roughness (i.e., S_a_ of ~24 μm for sample 24). According to the EDS analysis shown previously, these droplets could correspond to aluminum oxide. The morphological heterogeneity created as well as the surface condition can be quantified. It would be sufficient to determine the most homogeneous surface. 

**Figure 12:**
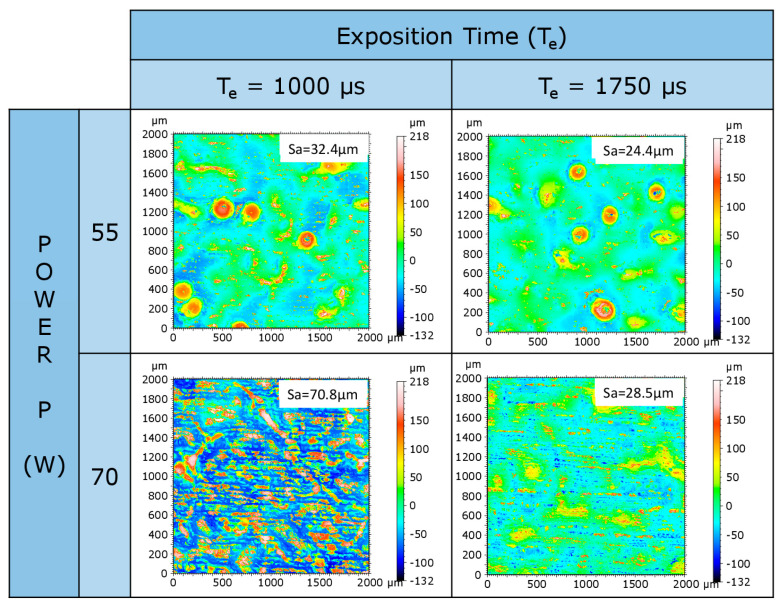
Topographic measures of extreme samples from N° 1 to N° 16.

The topographic analysis for the extreme condition of the samples from N° 1 to N° 16 is shown in Figure 12. The values obtained for the surface roughness (S_a_) varied between 70 and 24 µm.

### 3.2. Morphological Treatment

**Figure 13:**
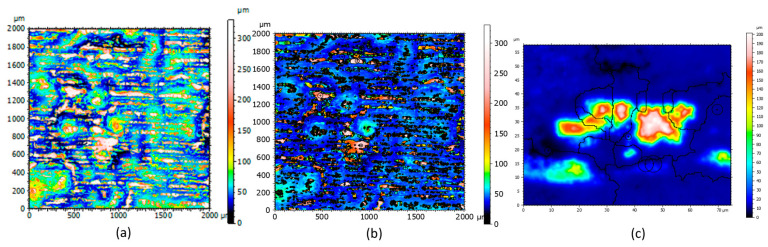
Topography measure of the sample N° 10: (**a**) amplitude map, (**b**) Motifs decomposition, and (**c**) zoom of motifs.

The morphological treatment will be explained on the topographical map of sample N° 10 (Figure 13a). 

An oriented zone appears visually. It seems to be constituted of clusters and laser scan way oriented. The measured step is 100 µm. Under the previously described clusters, a rippled structure emerges. To separate both structures, after a multi-scale analysis with different filters, a high-pass filter with 500 µm cut-offs enables a pretty good partition of the shape and the clusters. 

It is therefore clearly advisable to isolate these clusters and carry out a morphological analysis of their shapes rather than using conventional statistical indicators of roughness calculated overall image. To this end, Scott [32] created a method for segmenting a topographic image that is standardized in the field of surface topography [33]. This new segmentation method is now included in ISO 25178 as a method for discriminating significant peaks and valleys as a method for characterizing 3D patterns. This method is based on the application of a watershed algorithm combined with an algorithm for simplifying the graph of relations between particular points. An algorithm called Wolf pruning, allows sub-patterns to be gathered into significant patterns [34]. This peak merging is obtained by applying a threshold to motif heights concerning a height threshold, which is usually specified as a percentage of the total height of the map. This step is important for the calculation of the peak density, peak height, peak orientation, and peak curvature, etc. [35]. 

Then, the motif decomposition method is applied which allows for isolating the motifs. A Wolf Pruning threshold at 5% (the classical threshold used by default) permits the extraction of an elementary topography for each motif to gather them in a statistical description (Figure 13b,c). In some ways, this decomposition method performs a cluster grain size analysis of the topographical map.

**Figure 14:**
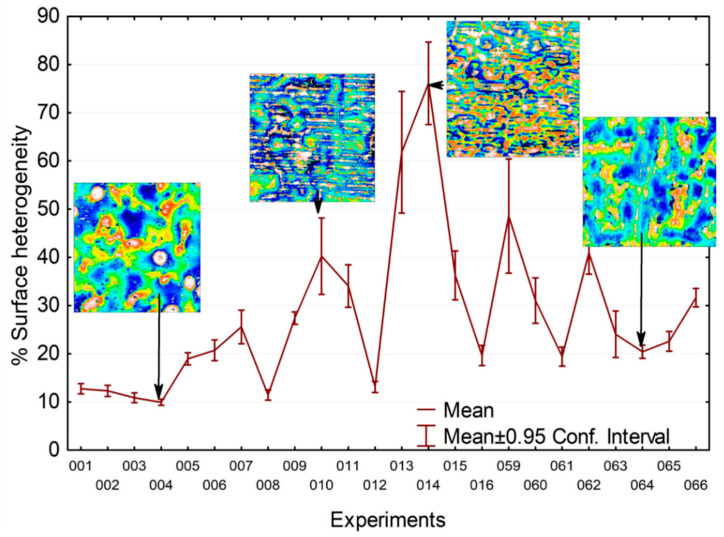
A graph of the percentage of surface heterogeneity for all samples of the experimental design showed in Table 1.

With the adaptation of the motif methods, only heterogeneous topographical zones are considered motifs. In other words, this algorithm was used for shape detection and classification. The total area of the clusters, characterized by the summation of all the elementary areas of each motif, can be calculated to obtain an acceptable cluster percentage by surface unity. This algorithm was applied on all topographical maps without any changes in morphological parameters to obtain the heterogeneity percentage, renamed surface heterogeneity (Figure 14). A smaller percentage means a more homogeneous surface. This percentage also highlights the lousy melting percentage of the powder in ink.

As five measurements were performed at random on all the surface coatings, the uncertainty of the surface heterogeneity coefficient can be determined. Figure 14 depicts, for all the experiments gathered in Table 1, the value of the surface heterogeneity coefficient and its relative uncertainty (confidence interval at 95% of the average). The graph shows a clear heterogeneity difference from 10% to 80% depending on the experiments. One can notice that the associate dispersion is relatively low for a low heterogeneity. However, substantial heterogeneity seems to imply a higher associate dispersion. It may show a poorer surface state control when the complete melting of the powder is not insured.

### 3.3. Statistical Treatments

Practically, the test plan of Table 1 is neither a factorial nor a pure fractional plan. Real effects of the melting process parameters (power, exposition time, distance between two pulses, and hatch space) on the quality of the surface must be treated with appropriate statistical tools. To analyze the effects of these four parameters mentioned above, we will use a General Linear Model (GLM) regression analysis using the statistical language SASTM (SAS Institute, North Carolina, USA). The Fisher random variable (F) value is used to determine the influence or non-influence of each process parameter. After processing statistical analysis, the following influences on each processing parameter for surface heterogeneity are obtained:

The most substantial influence comes from the power factor (P_w_) (F = 77, *p*-value < 0.00000);

Another one that influenced the surface heterogeneity strongly is the exposition time (te) (F = 48, *p*-value = 0.00001);

The distance between two pulses (d_p_) has a small influence on the surface heterogeneity (F = 6.9, *p*-value = 0.0002);

Surprisingly, the Hatch space (H_s_) has almost no statistical influence (F = 4.5, *p*-value = 0.035).

**Figure 15:**
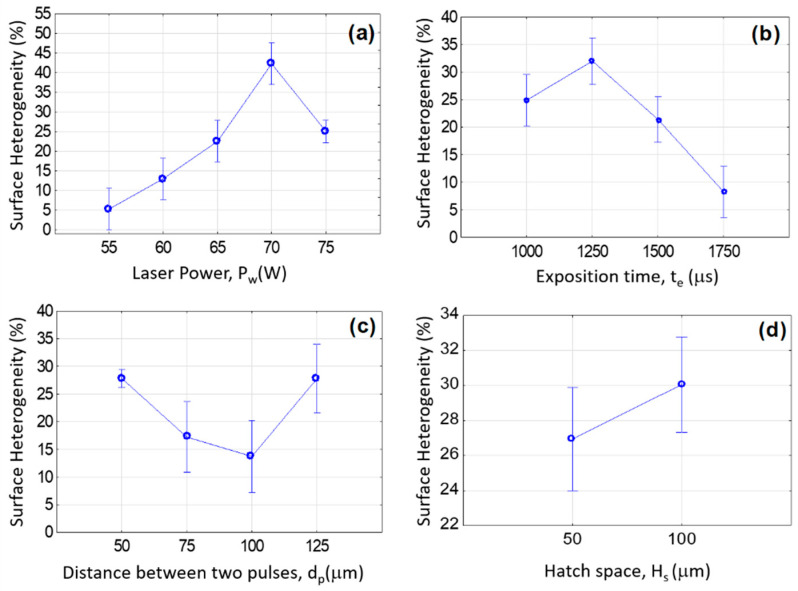
Statistical effects on the surface heterogeneity for different LM parameters. (**a**) The laser power effect, (**b**) the laser exposition time effect, (**c**) the distance between pulses, and (**d**) the hatch space.

Figure 15 depicts the evolution of the surface heterogeneity versus different values of the four LM process parameters P_w_, t_e_, d_p_, and H_s_ of the LM process (Table 1). The graphs (Figure 15) show the non-linearity of the process parameters P_w_, t_e_, and d_p_ associated with the 95% confidence intervals. The surface heterogeneity could be minimized if the melting operating parameters are optimized. After the optimization of the multi-varied equation obtained by GLM, the best LM parameter values to minimize surface heterogeneity are P_w_ = 55 W, t_e_ = 1750 µs, and d_p_ = 100 µm (the worst is P_w_ = 70 W, t_e_ = 1250 µs, d_p_ = 125 µm).

**Figure 16:**
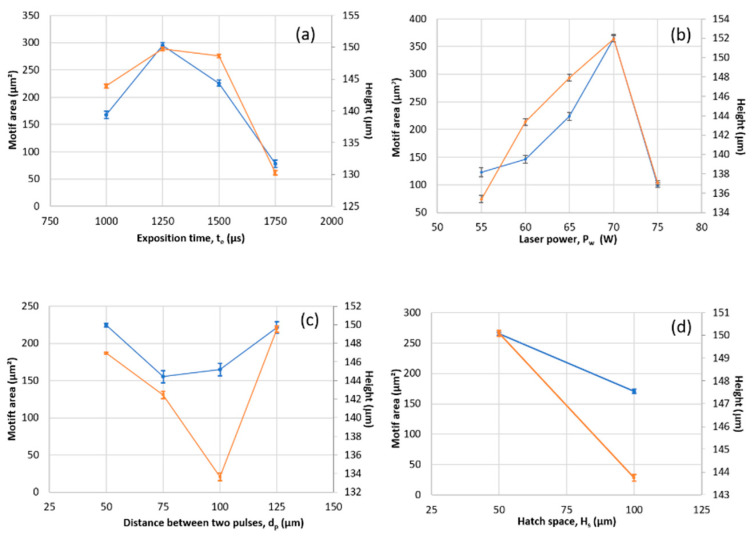
Results of the two variance analyses of all the process parameters: (**a**) Exposition time, (**b**) laser power, and (**c**) distance between two pulses and (**d**) Hatch space. Orange Curves represent the motif area and the blue curves represent the motif height.

Each motif corresponds to a particular geometrical shape. Thus, after a global analysis based on the area percentage of the motifs, it is safe to examine the morphology of each motif. Some morphological parameters are associated with each motif, such as its area, height, anisotropy, and curvature. Figure 16 shows the motifs’ area and height values for all experiments. These parameters are gathered in a morphological parameter vector. This way, for each morphological parameter, its empirical probability density can be estimated to include stochastic variation from the melting process. One of the most important morphological parameters representing the basis on which the motif morphology is defined is the motif equivalent diameter.

## 4. Discussion

**Figure 17:**
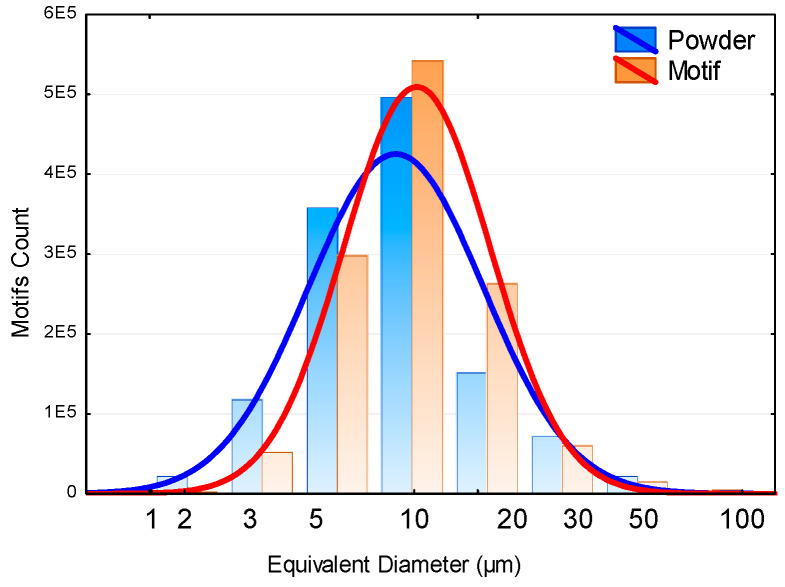
The number of motif counts vs. equivalent diameter.

The equivalent motif diameter histograms are depicted in Figure 17 (orange curve) with a logarithmic scale. This curve was established with almost 1.3 million motifs. The match between this repartition and a Gaussian law can be observed on the data with a logarithmic scale. It is equivalent to trying the match with a lognormal density law (red curve) [36]. The median particle size is highlighted, and its value is 10 µm (95% confidence interval). We used the logarithmic distribution to admit that the powder creation can be linked to a multiplicative process. Mitzenmacher [37] described these mechanisms by introducing the notion of power laws and proposed an interesting review of the origin of this law based on an information theory approach. The lognormal repartition of the equivalent diameter leads to thinking about classical particle repartition mechanisms. Smith and Jordan [38] proposed an interpretation of these parameters compared to the Gaussian laws and proved that this is an excellent mathematical model for particle size distribution. However, the physical processes are not introduced clearly. Applied to powder, authors justify the excellent fit of the diameter with the lognormal laws (on particle size in gas atomization of rapidly solidified aluminum powders [39], in SLM [40], and monocrystalline powder [41]. Even if the lognormal distribution is admitted in powder morphology [42,43], the physical processes that lead to the lognormal probability function are not explained. However, it remains a difficult task to explain the aims of this distribution. Smoluchowski [44] proposed that the lognormal law can be justified by coagulation and mass conservation, but the lognormal law is only obtained under certain conditions. More recently, Kiss et al. proposed a model for particle growth that can predict the well-known lognormal particle size distribution [45]. The basic idea is to suppose that the particle’s radius is “perfectly” linearly time-dependent (i.e., r ∝ t without Probability Density Function), and the time distribution is lognormal [46]. We have a program for the monte simulation proposed by Söderlund et al. [46] and find a good fit of our data with the model. 

However, it is essential to underline the lack of outliers. This homogeneous statistical distribution verifies the morphological method to segments by heterogeneous zones. A comparison between this distribution of motifs and the distribution of initial powders will be made. These analyses may highlight a potential link between the initial size of the particles introduced into the ink and the size of these clusters with the origin still undetermined. Figure 17 is the superimposition of the empirical probability density of powder diameter and the probability density associated with this diameter. A similarity between the powder diameter distribution and motif equivalent diameter repartition appears. There are fewer motifs for small equivalent diameters (>3 µm). Generally, extreme values tend to have a uniform repartition. This leads to a hypothesis: each motif corresponds to a non-melted ball. 

This counting method can be applied to powder metallurgy to test the melting homogeneity of the surface and, of course, it can be generalized to additive manufacturing (SLM, etc.).

**Figure 18:**
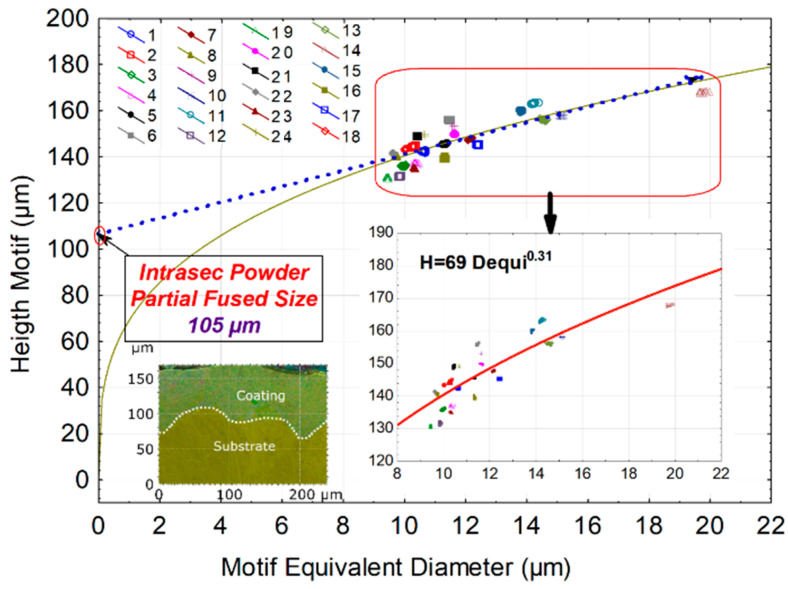
Graphics of height motif amplitude (H) versus motif equivalent diameter (D_equi_) of partial melting zones.

A physical interpretation (Figure 18) is now sought to create these non-homogeneous structures. Thus, for each experiment of the plan (24 experiments), the motif equivalent diameter (partial melting zones), and the median motif equivalent diameter are calculated. Then this average height (H) is plotted according to each experiment’s median diameter. 

Figure 18 highlights some phenomena: the height/diameter relationship is linear (linear line, blue dashed line). However, this link is not affined, which means a zero diameter does not correspond to a zero height. In a way, the geometric motif shapes are not homothety. To isolate the roughness peaks and show the laws of homothetic, one uses the motifs [47]. The motif decomposition method enables determining a gap in this homothetic. If the link diameter/motif analysis is less precise, a linear approximation is not the best statistical method to reproduce the function between height and diameter. Indeed, a power law describes this relation more precisely. The regression models were equal to H = 69 D_equi_^0.31^ (solid red line). This non-linearity, with an exponent smaller than unity, underlines a smaller increase in the pattern’s height as its diameter increases. This non-linearity leads to the deduction of an elementary physical phenomenon: the stability of the building in height cannot increase indefinitely with a mean diameter, whereas it will always be possible to create lateral clusters which will tend to increase the mean diameter by keeping the height more and more constant. Concretely, an increase in the equivalent diameter is related to the percolation of melting balls between them, increasing the average of all the unmelted balls slightly. 

By extending the linear approximation, we end up with a motif height of 105 μm (cf. red circle of Figure 18). This asymptotic value leads to the limited height of the pattern. This value of 105 μm is reached for a zero-equivalent diameter. It represents a melted height free of diameter. The physical interpretation implies a total absence of non-melted particles, so the melting of sprayed powders is completed. The coating thickness may be reached when all the balls are melted; in other words, the asymptotic value represents the thickness of the ideal homogeneous coating sprayed.

## 5. Conclusions

The use of Focus Variation microscopy has made it possible to investigate the highly rough surfaces produced by laser melting with sufficient precision to detect the physical mechanisms of powder melting. We have shown that a refined analysis of the three-dimensional roughness allows us to quantify the melting quality of powders by LM. For this purpose, a quantification method using the pattern method allowed the isolation of the heterogeneous zones. A dimensionless indicator bounded between 0 and 100 called “surface heterogeneity” was introduced, which allowed the determination of the optimal conditions of the manufacturing process parameters. The statistical analysis highlights the chemical segregation according to some process parameters that allowed for obtaining the best process parameters to develop the best surface morphology. 

Analyzing the statistical distribution of the equivalent diameters of the patterns, we obtain a lognormal probability density function identical to that of the particle size distribution of the powders used. By completing this description by the heights H, a power-law H = 69 D_equi_^0.31^ is found, showing a substantial deviation from the homothetic morphology involving fractal aspects due to the sphere-sphere aggregation of the powders during the melting process. A linearization of this fractal law allows us to obtain (in the limit d → 0) a Euclidean estimate of the surface corresponding to the thickness of the ideally smooth final coating, i.e., with a homogeneous total fusion of the powders by the LM process. Numerical simulations would validate this phenomenological modeling and describe the diffusion mechanisms encountered during fusion.

According to characterization results, the coatings obtained with a laser power of 75 W, dp of 50 μm, and exposition time of 1500 s presented higher hardness and fewer defects. Therefore, it seems that parameters such as laser power and distance between two pulses (dp) are the most influential on the properties and topography of the surface. It would be recommended in the next works to deposit coatings with the best conditions of morphological treatment obtained in this study, to validate this analysis.

## Figures and Tables

**Table 1:** Description of the samples according to the process parameters: Laser Power (P_w_), Hatch Space (H_s_), laser exposition time (t_e_), and distance between two pulses (d_p_).

N° of the Sample	1	2	3	4	5	6	7	8
P_w_ (W)	55	55	55	55	60	60	60	60
H_s_ (µm)	50	50	50	50	50	50	50	50
t_e_ (µs)	1000	1250	1500	1750	1000	1250	1500	1750
d_p_ (µm)	50	50	50	50	50	50	50	50
N° of the sample	9	10	11	12	13	14	15	16
P_w_ (W)	65	65	65	65	70	70	70	70
H_s_ (µm)	50	50	50	50	50	50	50	50
t_e_ (µs)	1000	1250	1500	1750	1000	1250	1500	1750
d_p_ (µm)	50	50	50	50	50	50	50	50
N° of the sample	17	18	19	20	21	22	23	24
P_w_ (W)	75	75	75	75	75	75	75	75
H_s_ (µm)	100	100	100	100	100	100	100	100
t_e_ (µs)	1250	1250	1250	1250	1500	1500	1500	1500
d_p_ (µm)	50	75	100	125	50	75	100	125

**Table 2:** Results of chemical composition by EDS and thickness of coatings deposited with H_s_ and d_p_ of 50 μm.

Sample	P_w_ (W)	t_e_ (s)	Chemical Composition (% at.)					Thickness (μm)
			Al	Cr	Mn	Fe	Mo	
1	55	1000	14.9 ± 3.3	19.5 ± 3.2	7.7 ± 2.0	46.1 ± 10.0	11.8 ± 1.7	34 ± 8
4	55	1750	15.1 ± 1.5	24.2 ± 1.7	12.9 ± 1.4	34.9 ± 4.0	12.9 ± 2.3	32 ± 5
5	60	1000	16.8 ± 1.7	22.3 ± 3.9	6.1 ± 1.5	41.1 ± 4.1	13.7 ± 0.9	51 ± 17
8	60	1750	15.9 ± 1.0	24.1 ± 1.3	12.8 ± 1.5	34.4 ± 1.3	12.7 ± 0.5	37 ± 9
9	65	1000	13.0 ± 2.2	17.5 ± 4.5	3.3 ± 0.9	53.6 ± 9.0	12.6 ± 2.6	40 ± 11
12	65	1750	11.3 ± 2.1	14.5 ± 1.2	9.1 ± 0.9	53.7 ± 6.2	11.4 ± 3.3	47 ± 9
13	70	1000	7.7 ± 1.9	9.7 ± 2.6	2.0 ± 0.8	74.4 ± 5.2	6.2 ± 1.7	90 ± 18
16	70	1750	10.9 ± 1.1	13.8 ± 1.4	2.6 ± 0.3	61.8 ± 1.7	10.9 ± 0.9	78 ± 32

**Table 3:** Results of chemical composition by EDS and thickness of coatings deposited with H_s_ of 100 μm and Pw of 75 W.

Sample	t_e_ (µs)	D_p_ (μm)	Al	Cr	Mn	Fe	Mo	Thickness (μm)
17	1250	50	13.4 ± 0.9	17.2 ± 0.8	4.5 ± 0.9	51.8 ± 3.0	13.1 ± 1.4	59 ± 6
20	1250	125	9.4 ± 1.7	12.1 ± 2.6	6.0 ± 0.8	65.6 ± 6.4	6.9 ± 1.5	60 ± 16
21	1500	50	11.5 ± 0.9	12.7 ± 0.6	3.4 ± 0.6	60.8 ± 2.7	11.6 ± 2.1	87 ± 17
24	1500	125	10.7 ± 0.6	13.7 ± 0.9	6.1 ± 0.4	60.7 ± 1.0	8.8 ± 0.3	79 ± 25
